# Mutational Analysis of *Eggplant Latent Viroid* RNA Circularization by the Eggplant tRNA Ligase in *Escherichia coli*

**DOI:** 10.3389/fmicb.2018.00635

**Published:** 2018-04-05

**Authors:** Teresa Cordero, Beltrán Ortolá, José-Antonio Daròs

**Affiliations:** Instituto de Biología Molecular y Celular de Plantas (Consejo Superior de Investigaciones Científicas – Universitat Politècnica de València), Valencia, Spain

**Keywords:** eggplant latent viroid, *Avsunviroidae*, tRNA ligase, RNA ligation, circular RNA, hammerhead ribozyme

## Abstract

*Eggplant latent viroid* (ELVd) is a relatively small non-coding circular RNA that induces asymptomatic infections in eggplants (*Solanum melongena* L.). Like other viroid species that belong to the family *Avsunviroidae*, ELVd contains hammerhead ribozymes in the strands of both polarities that self-cleave RNAs producing terminal 5′-hydroxyl and 2′,3′-cyclic phosphodiester groups. Available experimental data indicate that ELVd replicates in the chloroplasts of infected cells through a symmetric rolling-circle mechanism, in which RNA circularization is catalyzed by the chloroplastic isoform of the tRNA ligase. In this work, a mutational analysis was performed to gain insight into the sequence and structural requirements of the tRNA ligase-mediated circularization of ELVd RNAs. In the predicted minimum free energy conformation of the monomeric linear ELVd RNA intermediate of plus (+) polarity, the ligation site is located in the lower part of an opened internal loop, which is present in a *quasi*-rod-like structure that occupies the center of the molecule. The mutations analyzed herein consisted of punctual nucleotide substitutions and deletions surrounding the ligation site on the upper and lower strands of the ELVd *quasi*-double-stranded structure. Computational predictions of the mutated ELVd conformations indicated different degrees of distortions compared to the minimum free energy conformation of the wild-type ELVd linear monomer of + polarity. When these mutant RNAs were expressed in *Escherichia coli*, they were all circularized by the eggplant tRNA ligase with approximately the same efficiency as the wild-type ELVd, except for those that directly affected the ribozyme domain. These results suggest that the viroid ribozyme domains, in addition to self-cleavage, are also involved in the tRNA ligase-mediated circularization of the monomeric linear replication intermediates.

## Introduction

Viroids are a very unique type of plant infectious agents as they are exclusively constituted by a relatively small (246–401 nt) non-coding circular RNA. Despite the small size and with no help from self-encoded proteins, when these RNAs manage to enter compatible host cells they are able to move to appropriate cellular structures and to recruit the right host proteins to start their replication. Then the viroid progeny moves cell-to-cell through the plasmodesmata, the channels that traverse the cell walls of plant cells, and long-distance through the phloem to establish a systemic infection in the host plant frequently inducing a disease. The more than 30 viroid species known to date are classified into two families, *Pospiviroidae* and *Avsunviroidae*, depending on the presence of particular domains in the viroid molecule; more specifically a central conserved region (CCR) that is present in all members of the family *Pospiviroidae*, but is missing in those of the *Avsunviroidae*, and hammerhead ribozymes that are exclusive of this last family (Flores et al., 2015; Daròs, 2016b; Steger and Perreault, 2016; Brass et al., 2017; Giguère and Perreault, 2017).

Viroids replicate through an RNA-based rolling circle mechanism in which viroid circular RNAs serve as templates for reiterative transcription to produce multimeric RNAs of opposite polarity. Since viroids do not code for proteins, plus (+) polarity is arbitrarily assigned to the circular RNA strand that most abundantly accumulates in the host plant. Analyses of the replication mechanisms in different viroid species have indicated that members of both families follow two different variants of the rolling circle, termed asymmetric and symmetric (Branch and Robertson, 1984; Flores et al., 2008). *Potato spindle tuber viroid* (PSTVd), and possibly all members of the family *Pospiviroidae*, follow the asymmetric variant in which the minus (-) multimeric RNAs directly serve as templates for the transcription of multimeric + RNAs, which are then cleaved into monomeric linear viroid RNAs, and are finally circularized acquiring their mature form (Branch et al., 1988). In contrast, *Avocado sunblotch viroid* (ASBVd) and members of the family *Avsunviroidae* follow the symmetric variant in which the multimeric - RNAs self-cleave after the transcription through embedded hammerhead ribozymes, and the resulting monomers are ligated into monomeric circular RNAs. Next these viroid molecules, which are distinctive of this family, serve as templates in a second (symmetric) rolling circle for the transcription of multimeric + RNAs, which once again, are processed into monomeric circular RNAs of + polarity (Hutchins et al., 1985; Daròs et al., 1994).

*Potato spindle tuber viroid*, and apparently its family members, replicate in the nucleus of infected cells (Diener, 1971; Spiesmacher et al., 1983), while ASBVd and the other members of its family replicate in chloroplasts (Bonfiglioli et al., 1994; Navarro et al., 1999). However, things could be more intricate as *Eggplant latent viroid* (ELVd; family *Avsunviroidae*) is able to traffic from the cytoplasm to the nucleus and from there to the chloroplasts (Gómez and Pallás, 2012). Viroid RNA transcription is mediated by host DNA-dependent RNA polymerase II and chloroplastic nuclear-encoded polymerase (NEP), respectively, in viroids of the families *Pospiviroidae* and *Avsunviroidae* (Mühlbach and Sänger, 1979; Navarro et al., 2000). While multimeric replication intermediates of both polarities self-cleave through hammerhead ribozymes in viroids of the family *Avsunviroidae* (Flores et al., 2001), a host type-III RNase has been proposed to cleave the multimeric + transcripts of members of the *Pospiviroidae* by acting on transient double-stranded structures (Gas et al., 2007, 2008). Finally, viroid RNA circularization is catalyzed by host DNA ligase 1 and the chloroplastic isoform of the tRNA ligase in members of the families *Pospiviroidae* and *Avsunviroidae*, respectively (Nohales et al., 2012a,b).

*Eggplant latent viroid*, the only species in the genus *Elaviroid* (family *Avsunviroidae*) (Fadda et al., 2003; Daròs, 2017), induces asymptomatic infections in eggplants (*Solanum melongena* L.) and has been recently suggested to be a friendly experimental system to research many aspects of the *Avsunviroidae* molecular biology (Daròs, 2016a). Experiments done with this viroid have provided notable findings about hammerhead ribozyme activity (Carbonell et al., 2006), viroid replication (Nohales et al., 2012b; López-Carrasco et al., 2016), viroid movement (Gómez and Pallás, 2010, 2012) and viroid structure (Giguère et al., 2014; López-Carrasco et al., 2016). Regarding viroid circularization, a pioneering work showed that, despite not replicating in these cells, the dimeric ELVd transcripts expressed in the chloroplast of the unicellular green alga *Chlamydomonas reinhardtii* (phylum *Chlorophyta*) are efficiently processed to monomers and circularized (Molina-Serrano et al., 2007). Further work in *C. reinhardtii* chloroplasts showed that efficient ligation requires a *quasi*-double-stranded structure present in the central part of the molecule, which contains the ligation site in an internal loop (Martínez et al., 2009). A combination of *in vitro* and *in vivo* experiments indicated that the chloroplastic isoform of tRNA ligase (Englert et al., 2007) is the host enzyme involved in the circularization of ELVd and, most probably, of all the viroids in the family (Nohales et al., 2012b). *In vitro* experiments using a recombinant version of eggplant tRNA ligase produced in *Escherichia coli* have shown that, among several monomeric linear + ELVd RNAs, only those opened at the hammerhead ribozyme cleavage site are efficiently circularized (Nohales et al., 2012b). A circularization analysis in *E. coli* - ELVd does not replicate in these cells either - in which longer than unit ELVd + transcripts were co-expressed along with the eggplant tRNA ligase further supported that the domains of the viroid molecule outside the central *quasi*-double-stranded structure are dispensable for ligation (Daròs et al., 2014, 2018).

To gain further insight into the sequence and structural requirements of the tRNA ligase-mediated circularization of ELVd, we herein used the *E. coli* co-expression system to analyze the effect of the mutations surrounding the ligation site in this reaction. Surprisingly, all the assayed ELVd mutants were efficiently circularized by the eggplant tRNA ligase in *E. coli*, provided they did not directly affect the ribozyme domain, which supports the notion that this RNA domain, in addition to viroid RNA cleavage, is also involved in the circularization step that is mediated by the tRNA ligase.

## Materials and Methods

### Construction of Series of Plasmids to Express ELVd Mutants in *E. coli*

Plasmid pLELVd contains a longer-than-unit ELVd cDNA (from C327 to G46, GenBank accession number AJ536613; note that ELVd is circular and A333 is followed by G1), which included the repetition of the + -strand hammerhead ribozyme domain under the control of the *E. coli* murein lipoprotein promoter and the 5S rRNA (rrnC) terminator (Daròs et al., 2014, 2018). To induce a series of nucleotide substitutions and deletions in ELVd cDNA, this plasmid was employed as a template in polymerase chain reactions (PCR), for which the Phusion High-Fidelity DNA polymerase (Thermo Scientific) and different pairs of divergent primers were used (**Table 1**). Most of these pairs of primers, which harbored the desired mutations, contained a recognition site for type-IIS restriction enzyme *Bpi*I at the 5′ ends. The PCR products of full plasmid size were eluted after separation by electrophoresis in 1% agarose gels. Next they were digested with *Bpi*I (Thermo Scientific) and subjected to ligation with T4 DNA ligase (Thermo Scientific). In those cases in which primers did not harbor *Bpi*I sites (**Table 1**), deletions simply resulted from ligation of phosphorylated (T4 polynucleotide kinase, Thermo Scientific) blunt end PCR products. In both cases, *E. coli* DH5α were electroporated with the products of the ligation reaction and the recombinant clones selected on plates with Luria-Bertani (LB) medium that included ampicillin. The plasmids that contained the desired mutations were selected after sequencing (3130xl Genetic Analyzer, Life Technologies).

**Table 1:** Primers used to mutagenize the ELVd cDNA.

Mutant	Sequencebbb
C197A	5′-GGCGGAAGACGCTTTC***A***GACGGTGGGTTCGTCGAC-3′ 5′-CCGCGAAGACCGGAAAGTGTGTACTTTCCCTG-3′
G198U	5′-GGCGGAAGACGCTTTCC***T***ACGGTGGGTTCGTCGAC-3′ 5′-CCGCGAAGACCGGAAAGTGTGTACTTTCCCTG-3′
C200G	5′-GGCGGAAGACGCTTTCCGA***G***GGTGGGTTCGTCGAC-3′ 5′-CCGCGAAGACCGGAAAGTGTGTACTTTCCCTG-3′
G201A	5′-GGCGGAAGACGCTTTCCGAC***A***GTGGGTTCGTCGAC-3′ 5′-CCGCGAAGACCGGAAAGTGTGTACTTTCCCTG-3′
ΔU194-G204	5′-GGCGGAAGACGCCACTGGTTCGTCGACACCTCTCCC-3′ 5′-CCGCGAAGACCGAGTGTGTACTTTCCCTGATG-3′
ΔC197-G201	5′-GGCGGAAGACGCTTTCGTGGGTTCGTCGACACCTC-3′ 5′-CCGCGAAGACCGGAAAGTGTGTACTTTCCCTG-3′
ΔG1-U7	5′-TATGGGGCAGCGTTACAAGT-3′ 5′-GTGTGCCACCCCTGATGAGAC-3′
ΔG38-U42	5′-GACCTTTCGGTCTCATCAGG-3′ 5′-GGGGTTTCGCCATGGGTCGG-3′
ΔU47-G56	5′-CCCCATTTCGACCTTTCGGTC-3′ 5′-GGTCGGGACTTTAAATTCGG-3′
ΔG317-U326	5′-CTCTATCTCTCCTGGAAGGC-3′ 5′-CCCCATAGGGTGGTGTGTGC-3′

*bbb BpiI recognition and cleavage sites are on a gray background and underlined, respectively. The punctual nucleotide substitutions are in italics and bold.*

### ELVd and tRNA Ligase Co-expression in *E. coli*

*Escherichia coli* DH5α were co-electroporated with pLELVd (or derivatives with the different mutations) and p15LtRnlSm, a plasmid with a compatible p15A replication origin to express a recombinant version of the chloroplastic isoform of the eggplant tRNA ligase (GenBank accession no. JX0225157) under the control of the *E. coli* murein lipoprotein promoter and the rrnC terminator (Daròs et al., 2014, 2018). The recombinant clones that harbored both plasmids were selected on LB plates that contained ampicillin and chloramphenicol. To co-express the ELVd RNA and the tRNA ligase in *E. coli*, the colonies from these plates were grown in 2 ml of liquid LB medium that contained both antibiotics for 24 h at 37°C with vigorous shaking (225 revolutions per min). The K219A mutant of eggplant tRNA ligase was obtained by mutagenic PCR on template p15LtRnlSm using primers 5′- CAAGTGTGACCTCGACTATAG-3′ and 5′-CGCATTCTGGATCTCTTTTTATG-3′, as previously described for ELVd mutants.

### RNA Purification From *E. coli*

Cells in the 2-ml cultures were harvested by centrifugation and resuspended in 50 μl of TE buffer (10 mM Tris-HCl, pH 8.0, 1 mM EDTA). To break cells, one volume (50 μl) of a 1:1 mixture of buffer saturated phenol (1 M Tris-HCl, pH 8.0) and chloroform was added and the mix was vigorously vortexed. The aqueous phase that contained the *E. coli* RNA was recovered after centrifugation and was frozen.

### RNA Electrophoresis

Aliquots of 10 μl of the RNA preparations were mixed with one volume of loading buffer that contained 98% formamide, and was denatured by heating for 1.5 min at 95°C, followed by snap cooling on ice. After denaturation, RNA was separated by electrophoresis in 5% polyacrylamide gels (37.5:1 acrylamyde:*N,N’*-methylenebisacrylamide) that contained 8 M urea in TBE buffer (89 mM Tris, 89 mM boric acid, 2 mM EDTA). In some experiments, in order to further separate circular molecules, RNA preparations were subjected to a two-dimension electrophoresis. After separating the RNA as explained above, the whole lane was transversally laid on top of a second urea gel at a lower buffer concentration (0.25 × TBE) and electrophoresis continued.

### Northern Blot Hybridization Analysis

After electrophoresis, RNAs were electroblotted to positively charged nylon membranes (Nytran SPC, Whatman) and were cross-linked by irradiation with 1.2 J/cm^2^ UV light (254 nm, Vilber Lourmat). Hybridization was performed overnight at 70°C in 50% formamide, 0.1% Ficoll, 0.1% polyvinylpyrrolidone, 100 ng/ml salmon sperm DNA, 1% sodium dodecyl sulfate (SDS), 0.75 M NaCl, 75 mM sodium citrate, pH 7.0, with approximately 1 million counts per minute of a ^32^P-labeled ELVd probe of - polarity. The hybridized membranes were washed three times for 10 min with 2 × SSC, 0.1% SDS at room temperature and once for 15 min at 55°C with 0.1 × SSC, 0.1% SDS (the SSC buffer is 150 mM NaCl, 15 mM sodium citrate, pH 7.0). Hybridization signals were recorded by autoradiography using X-ray films (Fujifilm). The radioactive RNA probe consisted of a full-length ELVd RNA monomer of complementary polarity. This probe was obtained by *in vitro* transcription of a linearized plasmid with 20 U of T3 bacteriophage RNA polymerase (Roche) in 20-μl reactions that contained 40 mM Tris-HCl, pH 8.0, 6 mM MgCl_2_, 20 mM DTT, 2 mM spermidine, 0.5 mM each of ATP, CTP and GTP, and 50 μCi of [α-^32^P]UTP (800 Ci/mmol), 20 U RNase inhibitor (RiboLock, Thermo Scientific) and 0.1 U yeast inorganic pyrophosphatase (Thermo Scientific). Reactions were incubated for 1 h at 37°C. After transcription, the DNA template was digested with 20 U DNase I (Thermo Scientific) for 10 min at 37°C. The probe was purified by chromatography in a Sephadex G-50 column (mini Quick Spin DNA Columns, Roche).

### Prediction of RNA Secondary Structures

The Mfold algorithm (Zuker, 2003) was used to predict the minimum free energy conformation of the different monomeric linear ELVd RNAs using the default parameters^1^.

## Results

**FIGURE 1:**
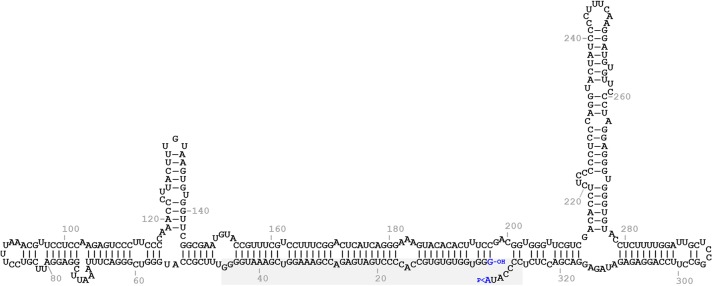
Predicted minimum free energy conformation of the monomeric linear ELVd replication intermediate of + polarity. The terminal nucleotides are highlighted in blue. The 5′-hydroxyl and 2′,3′-phosphodiester groups of the terminal nucleotides are indicated by -OH and >P, respectively. The domain of the + hammerhead ribozyme is on a gray background.

In the predicted conformation of minimum free energy of the monomeric linear + ELVd RNA (sequence variant AJ536613), the ligation site (positions 333-1) lays on the border of an internal opened loop in a long *quasi*-double-stranded structure that is present in the central part of the molecule (**Figure 1**). In this region, this predicted conformation mostly agrees on those of two independent experimental determinations of circular ELVd + RNAs obtained by high-throughput selective 2′-hydroxyl acylation analyzed by primer extension (hSHAPE) (Giguère et al., 2014; López-Carrasco et al., 2016). To study in-depth the sequence and structural requirements of the ELVd RNA circularization by the eggplant tRNA ligase, we used an *E. coli*-based co-expression system to analyze the effect of mutations on this loop. To avoid interference with the hammerhead ribozyme-mediated cleavage of the expressed ELVd longer-than-unit transcripts (a processing step prior to circularization), the first set of mutations were placed on the upper strand of the loop.

### Effect on Circularization of Punctual Substitutions That Change the Conformation of the Loop That Contains the ELVd Ligation Site

**FIGURE 2:**
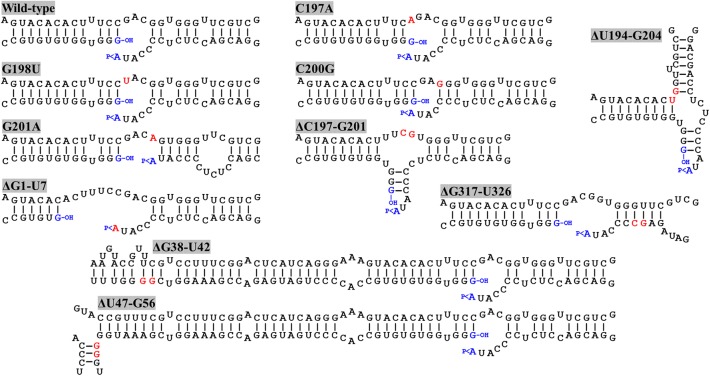
Predicted minimum free energy conformations of the regions around the ligation site of wild-type ELVd and different mutants. C197A, G198U, C200G, and G201A are punctual nucleotide substitution mutants, and ΔU194-G204, ΔC197-G201, ΔG1-U7, ΔG38-U42, ΔU47-G56, and ΔG317-U326 are deletions mutants. Nucleotide positions refer to ELVd sequence variant AJ536613. Mutated nucleotides and nucleotides on the border of deletions are highlighted in red. The terminal nucleotides ligated during circularization are in blue. The 5′-hydroxyl and 2′,3′-phosphodiester groups of the terminal nucleotides are indicated by –OH and >P, respectively.

**FIGURE 3:**
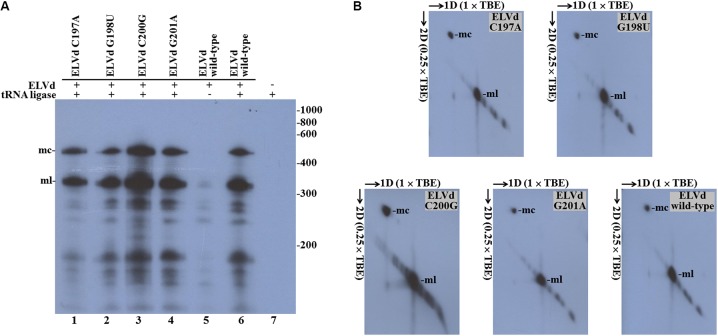
Eggplant tRNA ligase-mediated circularization of ELVd punctual substitution mutants in *Escherichia coli*. Total RNA from *E. coli* clones, in which longer-than-unit ELVd transcripts and tRNA ligase were co-expressed, were separated by **(A)** single denaturing PAGE in an 8 M urea, (×1) TBE gel or **(B)** two-dimension PAGE, first in an 8 M urea, (×1) TBE gel and second in an 8 M urea, (×0.25) TBE gel. ELVd + strands were detected by Northern blot analysis. **(A)** Lanes 1 to 4, *E. coli* clones in which ELVd mutants C197A, G198U, C200G and G201A were co-expressed with eggplant tRNA ligase; lanes 5 and 6, control clones in which wild-type ELVd was expressed alone or co-expressed with tRNA ligase, respectively; lane 7, control clone in which only tRNA ligase was expressed. The positions and sizes (in nt) of RNA markers are indicated on the right **(B)** The migration directions of RNA in both dimensions are indicated by arrows. **(A,B)** The positions of the monomeric circular (mc) and linear (ml) forms of ELVd are indicated.

By means of PCR with mutagenic primers, we created four different nucleotide substitutions on the upper strand of the loop where the ligation site is located on the ELVd RNA of + polarity. These four mutations were designed to alter the secondary structure around the ligation site. According to the Mfold prediction of minimum free energy conformations, substitution C197A opens the loop on the left-hand side, while G198U does not substantially change the structure (**Figure 2**). Substitution C200G closes the loop on the right-hand side, whereas G201A causes a strong restructuring of the whole region (**Figure 2**). The longer-than-unit ELVd transcripts that contained these mutations where co-expressed in *E. coli* along with the eggplant tRNA ligase. Total RNA was extracted from *E. coli* cells and separated by denaturing PAGE. Finally, ELVd + strands were detected by Northern blot hybridization using a complementary radioactive probe. Two prominent bands that apparently corresponded to the monomeric linear and monomeric circular ELVd RNAs of + polarity were detected in the lanes that corresponded to all four mutants (**Figure 3A**, lanes 1–4). The intensity of these bands did not differ from those in the wild-type ELVd control (**Figure 3A**, lane 6). The controls of this experiment also included the single expressions of the wild-type ELVd or the tRNA ligase. In these controls, *E. coli* cells were co-transformed with the corresponding empty expression plasmids. Only faint bands were detected when the tRNA ligase was not expressed (**Figure 3A**, lane 5), and no band was observed when ELVd RNA was not expressed (**Figure 3A**, lane 7). As previously shown, ELVd RNAs only accumulate efficiently in *E. coli* cells in the presence of eggplant tRNA ligase (Daròs et al., 2018). The same results were obtained in two additional replicates done of the whole experiment, in which independent *E. coli* clones were analyzed.

Of the two prominent bands, the lower (ml in **Figure 3A**) very precisely matches the expected position of the ELVd linear monomers of 333 nt, while the upper one (mc in **Figure 3A**) corresponds to a species that displays the electrophoretic behavior expected for the ELVd circular monomers, which must be delayed from the linear counterparts under denaturing conditions. To ensure that the upper bands corresponded to the ELVd circular monomers, equivalent aliquots of the above samples were separated by two-dimension denaturing PAGE at two different ionic strengths and the ELVd + strands were detected by Northern blot hybridization. As expected for circular RNA molecules, the slow migrating species further delayed in the second dimension at a low ionic strength, and deviated from the diagonal of the linear RNAs (**Figure 3B**). Taken together, these results indicate that the four assayed punctual nucleotide substitutions, which induced different alterations in the secondary structure of the loop on which the + ELVd ligation site is located have no effect on the eggplant tRNA ligase-mediated circularization.

### Effect on the Circularization of Deletions on the Upper Strand of the *Quasi*-Double-Stranded Domain That Contains the ELVd Ligation Site

**FIGURE 4:**
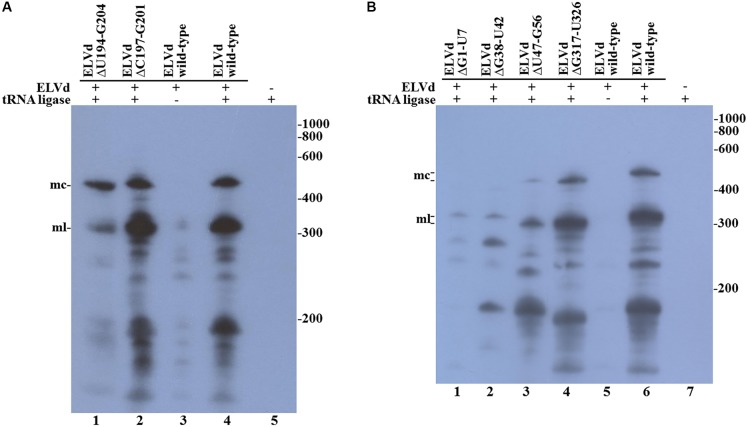
Circularization of ELVd deletions mutants by eggplant tRNA ligase in *E. coli*. Total RNA from *E. coli* clones, in which longer-than-unit ELVd transcripts and tRNA ligase were co-expressed, were separated by denaturing PAGE and transferred to a membrane for Northern blot hybridization of ELVd + strands. **(A)** Lanes 1 and 2, *E. coli* clones in which ELVd deletion mutants ΔU194-G204 and ΔC197-G201 were co-expressed with eggplant tRNA ligase; lanes 3 to 5, control *E. coli* clones in which wild-type ELVd, but not tRNA ligase, wild-type ELVd and tRNA ligase, and tRNA ligase, but not ELVd RNA, were, respectively, expressed as indicated. **(B)** Lanes 1 to 4, *E. coli* clones in which ELVd deletion mutants ΔG1-U7, ΔG38-U42, ΔU47-G56, and ΔG317-U326 were co-expressed with eggplant tRNA ligase; lanes 5 to 7, control *E. coli* clones in which wild-type ELVd alone, wild-type ELVd and tRNA ligase, and tRNA ligase alone were, respectively, expressed as indicated. **(A,B)** The positions and sizes (in nt) of RNA markers are indicated on the right, and the positions of the monomeric circular (mc) and linear (ml) forms of ELVd are indicated on the left of both panels.

We reasoned that the assayed punctual nucleotide substitutions may not have sufficiently altered the secondary structure of the loop that contains the ligation site to affect circularization. To test this hypothesis, two additional ELVd mutants were created, which consisted in the deletion of five (from position C197 to G201) or eleven (from position U194 to G204) nucleotides of the upper RNA strand. The Mfold prediction of the minimum free energy conformation of these deleted monomeric linear ELVd + RNA forms showed a profound alteration of the secondary structures around the ligation site in these two cases (**Figure 2**). The ELVd longer-than-unit transcripts that contained these deletions were co-expressed with eggplant tRNA ligase in *E. coli* and total RNA was extracted. RNA was separated by denaturing PAGE and the + ELVd strands were revealed by Northern blot hybridization. Two prominent bands, whose position was consistent with the migration of the monomeric linear and circular ELVd RNAs, were once again detected (**Figure 4A**, lanes 1 and 2). Like the above experiment, the intensity of these bands did not substantially differ from those in the control in which the wild-type ELVd was expressed (**Figure 4A**, lane 4). The same results were observed in the analysis of two additional independent *E. coli* clones of each mutant. These results indicate that the two assayed deletions, despite the strong impact on the secondary structure of the ligation domain, have no effect on the efficiency of the tRNA ligase-mediated circularization of ELVd.

### Effect on the Circularization of Deletions on the Lower Strand of the *Quasi*-Double-Stranded Domain That Contains the ELVd Ligation Site

Since punctual mutations and deletions on the upper strand of the *quasi*-double-stranded domain that contains the ligation site did not affect ELVd circularization, we assayed four deletions on the lower strand. Note that this strand contains the viroid + hammerhead ribozyme domain (**Figure 1**) and some of these deletions were expected to have a deleterious effect on the self-cleavage of the ELVd + RNA precursor. The two deletions that directly affected the ribozyme domain were the 7-nt deletion from G1 to U7 that completely eliminated the upper strand of the hammerhead helix I and the 5-nt deletion from G38 to U42 that eliminated a key hammerhead conserved motif. The 10-nt deletions from U47 to G56 and from G317 to U326 were created in both borders of the ribozyme domain (**Figure 2**). We co-expressed longer-than-unit transcripts containing these deletions with the eggplant tRNA ligase in *E. coli* and analyzed the ELVd + RNAs that accumulated in the bacteria by Northern blot hybridization. Bands corresponding to the monomeric linear ELVd + RNA were detected for all four mutants, but bands corresponding to monomeric circular ELVd + RNAs were only observed for deletion mutants not directly affecting the ribozyme domain (**Figure 4B**, compare lanes 1 and 2 with lanes 3 and 4). Same results were obtained in the analysis of two additional independent *E. coli* clones of each mutant.

**FIGURE 5:**
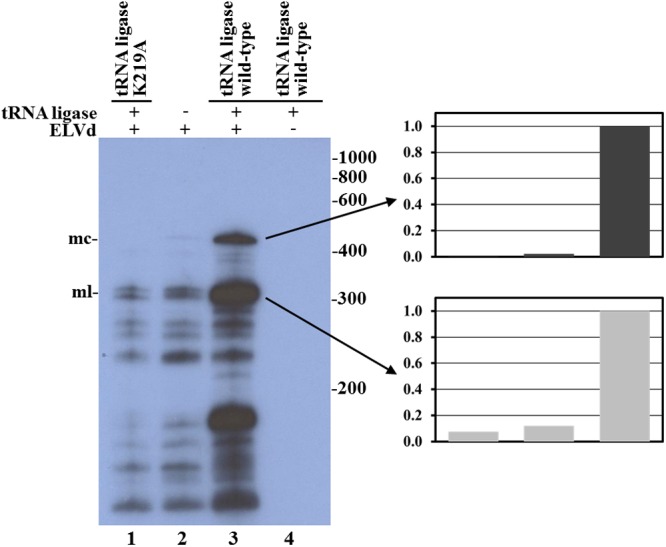
Analysis of eggplant tRNA ligase role in ELVd RNA circularization in *E. coli*. Northern blot hybridization analysis of total RNA from *E. coli* clones in which longer-than-unit ELVd transcripts were co-expressed with eggplant tRNA ligase. RNAs were separated by denaturing PAGE and transferred to a membrane. ELVd + strands were detected with a complementary ^32^P-labeled RNA probe. Lanes 1, 2, and 3, *E. coli* clones in which the wild-type ELVd was co-expressed with the K219A catalytic mutant of eggplant tRNA ligase (lane 1), no tRNA ligase (lane 2) or the wild-type tRNA ligase (lane 3). Lane 4, control clone in which the wild-type tRNA ligase was expressed alone. The positions and sizes (in nt) of RNA markers are indicated on the right of the panel, and the positions of the monomeric circular (mc) and linear (ml) forms of ELVd are indicated on the left. Normalized histograms of band intensities corresponding to monomeric circular and linear ELVd forms are presented on the right.

In view of these last results, we decided to rule out the possibility that, in our *E. coli* experimental system, the hammerhead ribozyme in addition to ELVd RNA cleavage was also catalyzing the RNA circularization. To do that, we compared the circularization of the wild-type ELVd transcript in the presence of the eggplant wild-type tRNA ligase and the K219A catalytic mutant. This mutation in a nucleotidyltransferase key lysine completely prevents the binding of an ATP molecule, which is critical for the function of different ATP-dependent ligases (Sawaya et al., 2003). By Northern blot hybridization analysis, we were unable to detect substantial amounts of ELVd circular monomers in bacteria that expressed the inactive tRNA ligase, contrary to what happens in bacteria that expressed the wild-type ligase (**Figure 5**, compare lanes 1 and 3). This result supports the notion that, despite the observed importance of the viroid hammerhead ribozyme domain, the catalytic activity that circularizes the ELVd RNA resides in the co-expressed eggplant tRNA ligase.

## Discussion

Viroids are non-coding RNAs capable of replicating and moving in the plants that they manage to infect. To do so, they must recruit host factors, such as RNA polymerases, RNases, nucleic acid ligases, RNA chaperones or RNA transporters, and take advantage of cellular structures to mediate the different steps of the infectious process. To design durable resistance strategies against viroid infection, we need to improve our knowledge about the molecular details that rule the interactions between viroid molecules and these host factors. One such host factor is the tRNA ligase, a conserved eukaryotic enzyme involved in the maturation of nuclear tRNAs. Intron-containing pre-tRNAs are cleaved by a tRNA splicing endonuclease that produce tRNA halves with 2′,3′-cyclic phosphodiester and 5′-hydroxyl ends, which are joined by the tRNA ligase using ATP (Englert and Beier, 2005; Phizicky and Hopper, 2015). Apart from the nucleus and cytoplasm, in plants this enzyme is located in chloroplasts (Englert et al., 2007), this being the replication site of the viroids that belong to the family *Avsunviroidae*, like ELVd (Flores et al., 2000). This enzyme ligates RNA molecules that contain 5′-hydroxyl and 2′,3′-cyclic phosphodiester ends, which are precisely the terminal groups produced by hammerhead ribozymes during the self-cleavage of the multimeric replication intermediates of these viroids. Indeed, a combination of *in vitro* experiments, in which a recombinant version of the chloroplastic isoform of the eggplant tRNA ligase was used, and *in vivo* experiments, in which the endogenous tRNA ligase from *Nicotiana benthamiana* was silenced, supported the notion that this is the enzyme involved in the circularization of the viroids that belong to the family *Avsunviroidae* during replication (Nohales et al., 2012b).

In infectious agents like viroids, where genetic information is so densely packed, an analysis of a particular step of the infection process by site-directed mutagenesis normally requires the use of an appropriate experimental system since mutations will most probably have side effects on other steps. These experimental systems aim to dissect the step of interest from the whole infectious process. In the past, we set up one of these systems to analyze the processing (cleavage and ligation) of the RNAs of the family *Avsunviroidae*. This system consisted of transplastomic clones of the unicellular green alga *C. reinhardtii* in which longer-than-unit viroid RNAs were expressed in the algal chloroplast (Molina-Serrano et al., 2007). With the viroids of the family *Avsunviroidae*, these RNAs self-cleaved and circularized rather efficiently in the algal chloroplast, but efficiency depended on particular viroid species (Molina-Serrano et al., 2007). In this system, in which viroids do not replicate, circularization is most probably mediated by the *C. reinhardtii* homolog of plant tRNA ligase. This system served to perform a mutational analysis of ELVd RNA processing, which supported that the hammerhead ribozyme domain is necessary and sufficient to mediate transcript cleavage. However, this analysis also indicated that during RNA circularization, other viroid parts were involved, most probably a *quasi*-double-stranded structure present in the central part of the molecule that contains the ligation site in an internal loop (Martínez et al., 2009). This result is consistent with a subsequent finding obtained with an *in vitro* circularization analysis of different ELVd monomeric forms using a recombinant version of the chloroplastic isoform of the eggplant tRNA ligase. Under those particular *in vitro* reaction conditions, this enzyme only circularized the genuine monomeric linear ELVd replication intermediate of + polarity, opened at the site which corresponded to the native hammerhead ribozyme. In contrast, five other monomeric linear ELVd RNAs opened at different sites along the molecule were not circularized, despite containing the same 5′-hydroxyl and 2′,3′-phosphodiester terminal groups (Nohales et al., 2012b).

Even though, the chloroplastic *C. reinhardtii* system having many interesting properties to study viroid processing, it also has some limitations. First, *C. reinhardtii* chloroplast transformation is labor-intensive and time-consuming. Second, and possibly most importantly, viroid ligation is catalyzed by an undefined enzyme in *C. reinhardtii* chloroplasts. To overcome these limitations, and once the host enzyme involved in viroid circularization was known, we set up a new experimental system that consisted of *E. coli* recombinant clones in which ELVd longer-than-unit transcripts and the chloroplastic isoform of the eggplant tRNA ligase were co-expressed. In these cells, ELVd transcripts self-cleaved to monomers, which were recognized by the eggplant tRNA ligase and efficiently circularized. However, no viroid RNA-to-RNA replication was detected (Daròs et al., 2014, 2018).

In the present work, we used this *E. coli*-based experimental system to further study the sequence and structural requirements of ELVd circularization by the eggplant tRNA ligase. We more specifically analyzed the effect of mutations on both strands of the central *quasi*-double-stranded structure that contains the ligation site in an internal loop in the predicted conformation of minimum free energy of the monomeric linear ELVd intermediate of + polarity (**Figure 1**). In the upper strand, we assayed four punctual nucleotide substitutions that have different effects on the secondary structure of this domain, as well as two deletions with a strong effect. In the lower strand, we assayed two deletions that directly affected the viroid ribozyme and only one of them had a strong effect on the secondary structure of the ligation domain, as well as two deletions outside the ribozyme domain, again with only one having a strong effect on the secondary structure of the ligation domain (**Figure 2**). Interestingly, both ELVd deletion mutants directly affected in the ribozyme domain were still able to self-cleave in *E. coli*, although with a low efficiency, most likely through the *trans*-complementation activity of the second wild-type ribozyme. Note that the + ELVd longer-than-unit transcripts expressed in *E. coli* consisted of viroid monomers flanked by two complete ribozymes. In these transcripts, the full-length viroid monomers (from G1 to A333) were preceded by ELVd nucleotides C327 to A333 and followed by ELVd nucleotides G1 to G46 to form two complete ribozymes that release the full-length monomeric linear intermediates.

Despite having more or less affected the secondary structure of the ligation domain, most of the ELVd mutants analyzed in this work (C197A, G198U, C200G, G201A, ΔU194-G204, ΔC197-G201, ΔU47-G56, and ΔG317-U326) were efficiently circularized by the eggplant tRNA ligase in *E. coli*, except for those (ΔG1-U7 and ΔG38-U42) that directly affected the ribozyme domain (**Figures 3**, **4**). With the only concern that the precursors of these two mutants self-cleave in *E. coli* with low efficiency, due to mutations in one of the ribozymes, lack of circularization of the corresponding monomeric linear intermediates suggests the hypothesis that the eggplant tRNA ligase may not recognize the conformation of minimum free energy, rather a hypothetical transient structure formed by the lower strand of ELVd. This hypothesis would reconcile previous *in vitro* results in which + ELVd monomers opened at sites different to that which corresponded to the hammerhead ribozyme were not circularized by a purified eggplant tRNA ligase (Nohales et al., 2012b). The hammerhead ribozyme fold is just one of the many potential transient structures that the lower strand of the *quasi*-double-stranded structure can adopt. However, if this would finally be the physiological ligation fold, the experimental results presented herein clearly indicated that viroid circularization is not the result of a backward ribozyme reaction (Nelson et al., 2005; Canny et al., 2007) because no substantial circularization was detected when the eggplant tRNA ligase was not expressed in *E. coli* (**Figures 3**, **4**) or when a ligase catalytic mutant was expressed (**Figure 5**). A deeper analysis with additional mutants in the ribozyme domain will be necessary to shed some more light about the contribution of the viroid hammerhead ribozyme domain in ELVd circularization.

## Author Contributions

J-AD conceived the work and designed the experiments in close collaboration with TC and BO. TC and BO performed the experiments. TC, BO, and J-AD analyzed the data. J-AD wrote the manuscript with inputs from TC and BO. All authors read and approved the final manuscript.

## Conflict of Interest Statement

The authors declare that the research was conducted in the absence of any commercial or financial relationships that could be construed as a potential conflict of interest.

## Footnotes

1 http://unafold.rna.albany.edu/?q=mfold/RNA-Folding-Form
